# Avellanin A Has an Antiproliferative Effect on TP-Induced RWPE-1 Cells via the PI3K-Akt Signalling Pathway

**DOI:** 10.3390/md22060275

**Published:** 2024-06-13

**Authors:** Chang Xu, Guangping Cao, Hong Zhang, Meng Bai, Xiangxi Yi, Xinjian Qu

**Affiliations:** 1 Faculty of Pharmacy/Institute of Marine Drugs, Guangxi University of Chinese Medicine, Nanning 530200, China; xuchang2022@stu.gxtcmu.edu.cn (C.X.); cao15765981927@163.com (G.C.); zhanghong2023@stu.gxtcmu.edu.cn (H.Z.); 2 Guangxi Key Laboratory of Marine Drugs, Guangxi University of Chinese Medicine, Nanning 530200, China

**Keywords:** marine organisms, *Aspergillus fumigatus*, benign prostatic hyperplasia, avellanin A

## Abstract

Cyclic pentapeptide compounds have garnered much attention as a drug discovery resource. This study focused on the characterization and anti-benign prostatic hyperplasia (BPH) properties of avellanin A from *Aspergillus fumigatus* fungus in marine sediment samples collected in the Beibu Gulf of Guangxi Province in China. The antiproliferative effect and molecular mechanism of avellanin A were explored in testosterone propionate (TP)-induced RWPE-1 cells. The transcriptome results showed that avellanin A significantly blocked the ECM–receptor interaction and suppressed the downstream PI3K-Akt signalling pathway. Molecular docking revealed that avellanin A has a good affinity for the cathepsin L protein, which is involved in the terminal degradation of extracellular matrix components. Subsequently, qRT-PCR analysis revealed that the expression of the genes *COL1A1*, *COL1A2*, *COL5A2*, *COL6A3*, *MMP2*, *MMP9*, *ITGA2*, and *ITGB3* was significantly downregulated after avellanin A intervention. The Western blot results also confirmed that it not only reduced ITGB3 and FAK/p-FAK protein expression but also inhibited PI3K/p-PI3K and Akt/p-Akt protein expression in the PI3K-Akt signalling pathway. Furthermore, avellanin A downregulated Cyclin D1 protein expression and upregulated Bax, p21^WAF1/Cip1^, and p53 proapoptotic protein expression in TP-induced RWPE-1 cells, leading to cell cycle arrest and inhibition of cell proliferation. The results of this study support the use of avellanin A as a potential new drug for the treatment of BPH.

## 1. Introduction

Benign prostatic hyperplasia (BPH), which refers to the prostate transition zone and periurethral hyperplasia of epithelial and fibromuscular tissue growth, is one of the most common urological diseases in middle-aged and older men worldwide, and its incidence gradually increases with age [1]. At present, BPH is mainly treated with drugs such as 5α-reductase inhibitors and α blockers. Although both antagonists are effective in the treatment of BPH, these drugs have many adverse side effects, such as abnormal ejaculation, erectile dysfunction, and gynaecomastia [2]. Therefore, to more effectively prevent and treat BPH, improve the quality of life of patients, and reduce the adverse reactions caused by drugs, finding new targets and developing effective drug candidates with fewer adverse reactions are hot research directions [3].

The growth of marine microorganisms in a special environment produces a variety of secondary metabolites with special chemical structures [4]. Many secondary metabolites have a variety of pharmacological effects, such as antibacterial, anti-inflammatory, anti-tumour, or antiviral effects, so they have received extensive attention and application [5,6,7]. As a result, marine microbial secondary metabolites have become a very promising source of drug candidates [8]. Mangrove plants mainly grow in tropical and subtropical intertidal zones and are an important part of coastal wetland ecosystems, which are of great significance in terms of their ecological and economic value [9]. As endophytic fungi in mangrove plants have become a new hotspot for drug research and development, in recent years, many scientific research groups around the world have carried out research on the secondary metabolites of mangrove endophytic fungi and obtained a series of compounds with novel biological activities such as anti-inflammatory, anti-tumour, antibacterial, or antiviral effects [10].

Naturally occurring cyclic peptides have garnered much attention as a drug discovery resource [11,12]. This is because cyclic peptides are composed of amino acid residues, including nonproteinogenic residues, which are arranged in a three-dimensional structure and have high affinity for their target biomolecules [12]. In addition, cyclic peptides are reported to be more stable and membrane-permeable than general linear peptides [13].

**Figure 1:**
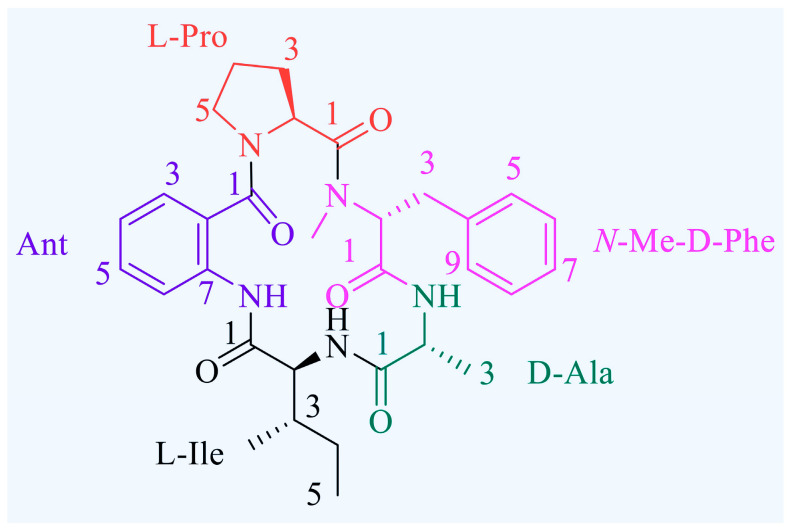
Avellanin A structure.

Avellanin A (C_31_H_39_N_5_O_5_, *m*/*z*: 561.2951) is a cyclic pentapeptide compound, which was first found in *Hamigers avellanea* [14]. We report here the re-isolation of this compound from *Aspergillus fumigatus* fungus in marine sediment samples collected in the Beibu Gulf of Guangxi Province in China. Avellanin A was obtained as a white powder, and HRESIMS analysis gave a pseudomolecular ion [M + H]^+^ at *m*/*z* 562.3024, corresponding to the molecular formula of C_31_H_39_N_5_O_5_ (*Δ* −0.05 mmu for C_31_H_40_N_5_O_5_; Figure S1). To date, the biological activity associated with avellanin A has only inhibited apoB in HepG2 cells without exhibiting cytotoxicity, and other biological activities need to be further explored [15]. Avellanin A is a cyclic pentapeptide compound linked in the order Ant-L-Pro-D-Ala- N-Me-D-Phe-L-Ile (Figure 1, Figures S2 and S3 and Table S1). The present study was designed to further explore the antiproliferative effects of avellanin A on RWPE-1 cells, ultimately revealing that this novel candidate therapeutic agent may inhibit PI3K-Akt pathway activity, suggesting that it may be useful as a new option for treating BPH patients.

## 2. Results

### 2.1. Antiproliferative Efficacy of Avellanin A on RWPE-1 Cells

Initially, the antiproliferative efficacy of avellanin A was investigated by treating it with testosterone propionate (TP)-induced RWPE-1 cells and performing a CCK-8 analysis. 

**Figure 2:**
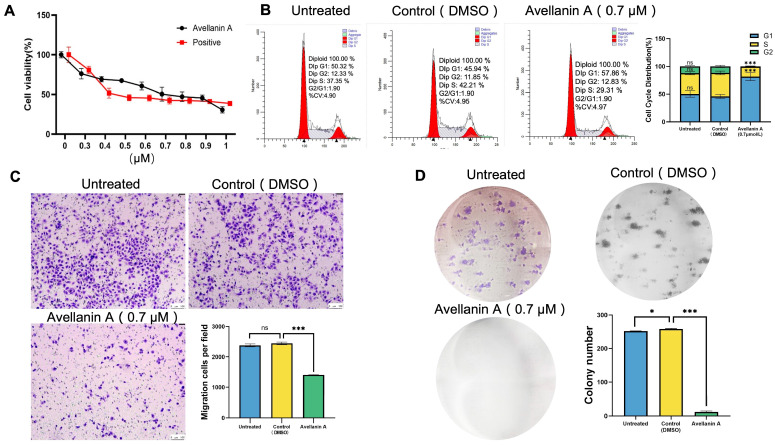
Antiproliferative efficacy of compound avellanin A on RWPE-1 cells: (**A**) RWPE-1 cells were treated with the indicated concentrations of compound avellanin A for 48 h, and the cell viability was determined by a CCK-8 assay. (**B**) The RWPE-1 cells were treated with indicated concentrations of compound avellanin A for 48 h, and the cell cycle distribution was assessed by flow cytometric analysis; ns, not significant, *** *p* < 0.001, compared to the control group. (**C**) Transwell assay evaluating the migration abilities of RWPE-1 cells treated with the indicated concentrations of compound avellanin A for 7 days. ns, not significant, *** *p* < 0.001, compared to the control group. (**D**) RWPE-1 cells were treated with the indicated concentrations of compound avellanin A for 7 days, after which colony formation was assessed; * *p* < 0.05 and *** *p* < 0.001, compared to the control group.

This approach revealed that avellanin A strongly inhibited RWPE-1 cell proliferation after treatment for 48 h in a dose-dependent manner. The avellanin A IC_50_ values for the RWPE-1 cell lines were calculated to be 0.72 µM (Figure 2A). 

The proliferation of normal cells is dependent on the cell growth cycle. To detect the impact of avellanin A on the cell cycle, the RWPE-1 cells were treated with avellanin A (0.7 μM) for 48 h. This process clearly showed that most of the RWPE-1 cells treated with avellanin A were arrested in the G1 phase, which inhibited the cell cycle progression (Figure 2B). Furthermore, Transwell assays were used to examine the effect of the drugs on cell migration ability. Compared with the control group, the number of transmembrane cells in RWPE-1 cells treated with avellanin A was significantly reduced, which confirmed that avellanin A significantly inhibited RWPE-1 cell migration (Figure 2C). In addition, the observations of colony formation assays demonstrated that avellanin A had the most potent inhibitory effect on RWPE-1 cell growth in a concentration-dependent manner (Figure 2D).

### 2.2. Transcriptome Analysis of RWPE-1 Cells Treated with Avellanin A

**Figure 3:**
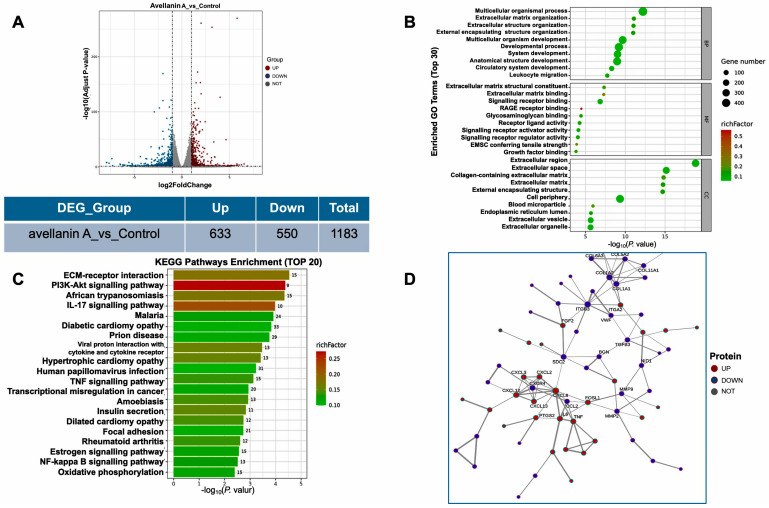
Transcriptome analysis of RWPE-1 cells treated with compound avellanin A vs. the control: (**A**) Volcanic map showing the differential gene expression distribution. The scattered dots in the figure represent individual genes, with grey dots indicating genes with no significant differences, red dots representing upregulated genes with significant differences, and blue dots representing downregulated genes with significant differences. Statistics of differentially expressed genes. A total of 1183 differentially expressed genes were identified, including 633 upregulated genes and 550 downregulated genes. (**B**) GO functional enrichment analysis of the DEGs. (**C**) KEGG pathway enrichment of differentially expressed genes. (**D**) PPI network construction for the identification of hub genes.

To investigate the mechanism of action of compound avellanin A on RWPE-1 cells, we conducted RNA-seq. After RWPE-1 cells were treated with the compound avellanin A at a concentration of 0.7 μM for 48 h, transcriptome analysis revealed that the heatmap showed a clear trend in the clustering of genes expressed in RWPE-1 cells between the compound avellanin A group and the control group (Figure 3A). A total of 1183 differentially expressed genes (DEGs) were identified, 633 of which were significantly upregulated and 550 of which were significantly downregulated.

Gene Ontology (GO) is a standardised functional classification system that describes the properties of genes and gene products in organisms in three aspects: biogenesis-involved biological process (BP), molecular function (MF), and cellular component (CC). The results of GO analysis can visually show the overall functional enrichment characteristics of all differentially expressed genes, and these significantly enriched genes are related to core biological functions. The enrichment of GO results indicated that avellanin A had a significant effect on cellular components (Figure 3B), including the extracellular space, collagen-containing extracellular matrix, extracellular matrix, and external encapsulating structure.

Moreover, KEGG pathway analysis was conducted to identify the pathways involved. The enrichment results for the differentially expressed genes related to metabolic pathways indicated that the compound avellanin A had a significant effect on cellular life processes, substance transport, and metabolic pathways (Figure 3C). The analysis revealed that the differentially expressed genes were primarily enriched in several pathways, notably ECM–receptor interaction, the PI3K-Akt signalling pathway, the IL-17 signalling pathway, malaria, and diabetic cardiomyopathy. Through KEGG analysis, the top 20 pathways were identified, among which many of the enriched genes were associated with the ECM-related signalling pathway (Figure 3D). 

Subsequently, PPI network analysis (Figure 3D) revealed that avellanin A significantly affected the expression of genes associated with the extracellular matrix (ECM), such as genes in the collagen subfamily (*COL1A1*, *COL1A2*, *COL5A2*, *COL6A3*), integrin subfamily (*ITGB3*, *ITGA2*), and MMP subfamily (*MMP2*, *MMP9*), as well as genes encoding FGF2 and VWF, which were significantly downregulated after avellanin A intervention. Therefore, bioinformatics results from transcriptome sequencing suggest that avellanin A may inhibit the expression of extracellular matrix integrins, collagen, and matrix metalloproteinases, thereby inhibiting the expression of the downstream PI3K-Akt signalling pathway and thereby inhibiting the progression of TP-induced BPH.

### 2.3. Interaction of Avellanin A with the Cathepsin L

**Figure 4:**
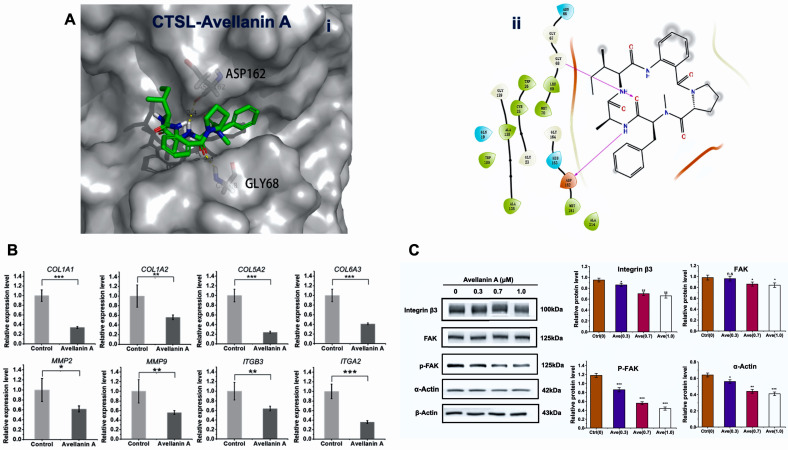
The binding mode of avellanin A to its targets, determined by molecular docking: (**A**) Binding mode of avellanin A to cathepsin L: (**i**) three-dimensional structures of the binding pockets were constructed with PyMOL 3.0 software; (**ii**) 2D interactions of compounds and their targets. (**B**) Relative mRNA expression of *COL1A1*, *COL1A2*, *COL5A2*, *COL6A3*, *MMP2*, *MMP9*, *ITGA2,* and *ITGB3*; * *p* < 0.05, ** *p* < 0.01, and *** *p* < 0.001, compared to the control group. (**C**) The expression of ITGB3, FAK/p-FAK, and α-Actin was analysed by Western blotting. The analysis utilized β-Actin as a reference for quantifying relative gene expression levels. * *p* < 0.05, ** *p* < 0.01, and *** *p* < 0.001, compared to the control group.

To evaluate the affinity of avellanin A for its targets, we performed molecular docking analysis. According to the docking results with the cathepsin L, avellanin A interacts with the basic amino acids Gly68 and Asp162 of the cathepsin L protein through hydrogen bonding and has a low binding energy of −6.2 kcal/mol, indicating highly stable binding (Figure 4A). 

The protein cathepsin L encoded by this gene is a lysosomal cysteine proteinase that plays a major role in intracellular protein catabolism. Its substrates include collagen and integrin. Next, using fluorescence quantitative PCR analysis, it was revealed that the expression of the genes *COL1A1*, *COL1A2*, *COL5A2*, *COL6A3*, *MMP2*, *MMP9*, *ITGA2,* and *ITGB3* was significantly downregulated after avellanin A intervention (*p* < 0.05, *p* < 0.01; Figure 4B). Furthermore, Western blot experiments confirmed that the protein expression of ITGB3 and FAK/p-FAK was reduced after avellanin A intervention in TP-treated RWPE-1 cells (Figure 4C and Figure S4). Thus, we hypothesised that avellanin A may inhibit the activity of the lysosomal cysteine proteinase cathepsin L, decrease the expression of extracellular matrix components, and decrease FAK/p-FAK expression via the downstream PI3K-Akt signalling pathway, leading to cell cycle arrest and inhibition of cell proliferation.

### 2.4. Avellanin A Suppressed the PI3K-Akt Pathway in RWPE-1 Cells

The PI3K/Akt pathway is significantly associated with increased proliferation and survival. To verify whether PI3K-Akt signalling might mediate avellanin A function in RWPE-1 cells, Western blotting was used to analyse changes in the expression of proteins involved in the PI3K-Akt signalling pathway. 

**Figure 5:**
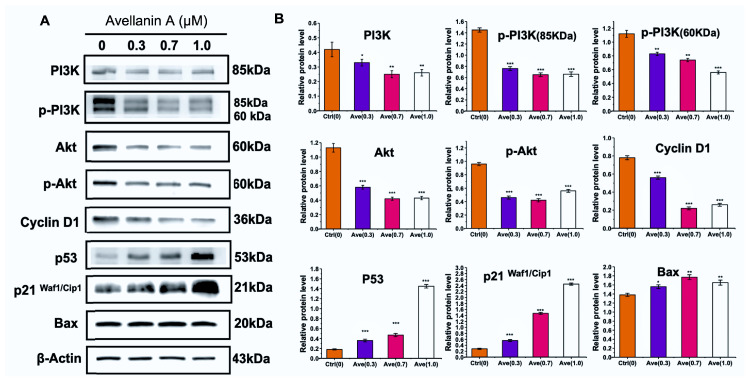
Effect of avellanin A on PI3K-Akt pathway-related protein expression in RWPE-1 cells. (**A**) The p-PI3K/PI3K, p-Akt/Akt, p53, Cyclin D1, P21^Waf1/Cip1^, and Bax proteins were analysed by Western blotting. The analysis utilized actin as a reference for quantifying relative protein expression levels. (**B**) Relative quantitative detection of protein expression compared with control. * *p* < 0.05, ** *p* < 0.01, and *** *p* < 0.001, compared to the control group.

As shown, the PI3K protein expression levels decreased in RWPE-1 cells with increasing avellanin A concentrations; the PI3K and phosphorylated PI3K protein levels were decreased. Moreover, the total Akt protein and phosphorylated Akt protein levels were decreased significantly in control and treated RWPE-1 cells, which confirmed the inhibition of the PI3K-Akt pathway by avellanin A (Figure 5A and Figure S5). To further understand the function of avellanin A in cell proliferation and apoptosis, we assessed the expression of several proteins involved in the Akt pathway by Western blotting. The results showed that avellanin A significantly decreased the expression of cyclin D1 compared with that in the control group (*p* < 0.01). Conversely, there was a significant increase in the protein expression of the apoptosis markers Bax, p21^Waf/Cip1^, and P53 (*p* < 0.01) in RWPE-1 cells as the avellanin A concentration increased (Figure 5B). These findings suggest that avellanin A may modulate the progression of BPH by affecting the cell cycle and blocking the division and replication of RWPE-1 cells through the PI3K-Akt pathway.

## 3. Discussion

Mangrove plants mainly include plants such as Red Sea olive plants, autumn eggplant plants [16], and so on. There is a long history of research on mangroves, and a variety of drugs for the treatment of human diseases have been extracted from mangrove plants [15]. By the end of 2023, 1565 compounds with new structures had been isolated from mangrove endophytic fungi, of which 613 compounds had broad-spectrum biological activity, providing guidance for new drug development [17].

In this project, we studied the molecular mechanism of the inhibitory activity of avellanin A from *Aspergillus fumigatus* GXIMD 03099, which is an endophytic fungus in mangrove plants, to provide ideas for the development of new drugs for the treatment of BPH. Avellanin A was originally isolated from *Hamigera avellanea*. The total synthesis of this compound was subsequently completed in 1989 and 2021 [14]. Previous reports have demonstrated that it inhibits apolipoprotein B production in HepG2 cells without exhibiting cytotoxicity. To date, no other biological activities of avellanin A have been reported.

To investigate the effect of avellanin A on RWPE-1 cells, we conducted RNA-seq, and subsequent KEGG analysis found that avellanin A significantly affected ECM–receptor interactions and their downstream signalling pathways. Furthermore, molecular docking revealed that avellanin A has a good affinity for the cathepsin L protein. Cathepsin L is widely distributed in the lysosomes of mammals [18]. Cathepsin L is a lysosomal cysteine proteinase that is secreted to dissolve intracellular and endocytic proteins. Under normal circumstances, cathepsin L performs biological functions in lysosomes [19]. However, changes in the expression levels and lysosomal state cause a portion of cathepsin L to be secreted into the extracellular environment, which is involved in the terminal degradation of extracellular matrix components such as collagen and elastin [20]. Studies have shown that the downregulation of cathepsin L inhibits the proliferation, invasion, and migration of glioma cells [21]. Moreover, in vitro experiments revealed that cathepsin L overexpression promoted proliferation, migration, and invasion in MCF-7 and MDA-MB-231 cells, while cathepsin L knockdown decreased proliferation, migration, and invasion in MDA-MB-231 cells [22]. Moreover, previous studies reported that cathepsin L can promote tumour cell proliferation by activating CCAAT-displacement protein/cut homeobox (CDP/Cux) transcription factors and accelerating entry into the S phase of the cell cycle [23]. In this study, avellanin A was shown to inhibit TP-induced prostate cell proliferation by halting the G1 phase of the cell cycle. It is speculated that the reason may be that avellanin A binds to cathepsin L and inhibits its activity, resulting in a decrease in the expression of ECM components such as collagen and integrin proteins, reducing the transduction of downstream signalling pathways, and ultimately inhibiting cell proliferation, survival, and migration.

The adhesion pathway kinase FAK plays a central role in intracellular communication. Activation of the focal adhesion kinase FAK and overexpression of focal adhesion proteins lead to abnormal adhesion of the extracellular matrix to epithelial cells [24], which is essential for the development of BPH [25]. A study showed that the PI3K/Akt signalling pathway is activated by interactions with the ECM, which participates in the cell proliferation process [26,27]. The activation of the PI3K signalling pathway phosphorylates AKT, increasing cell survival and decreasing apoptosis [28]. Significantly elevated levels of phosphorylated AKT and PI3K gene expression were observed in prostate tissue from BPH patients [29]. Our results revealed that FAK and the PI3K/Akt signalling pathway were downregulated in RWPE-1 cells after avellanin A intervention, significantly reducing cell survival.

Furthermore, these interferences are most likely achieved downstream of the PI3K-AKT signalling pathway. p21^Waf/Cip1^ functions as a cell cycle inhibitor and antiproliferative effector and plays an important role in controlling the cell cycle by binding to multiple Cyclin/CDK complexes of multiple phases [30,31]. Here, the protein level of p21^Waf/Cip1^ increased markedly. Consistent with the above results, the protein levels of cyclin D1 decreased significantly. Cyclin D1 is recognized as a crucial indicator of cell proliferation and serves as a pivotal regulator of cell cycle checkpoints governing G2/M and G1/S transitions [32,33]. Upon cell cycle initiation, cyclin D1 is rapidly upregulated, facilitating the division and replication of prostate stromal and epithelial cells [34,35]. Cyclin D1 functions as a vital sensor and integrator of extracellular signals in normal physiological processes, modulating cellular activities by binding to cyclin-dependent kinases [36]. p53 plays an important role in many parts of the cell cycle, and when the chromosomal DNA of a cell is damaged in the G1 phase, p53 transcriptional activity is enhanced, which induces the activation of the *p21^Waf/Cip1^* gene, which in turn causes p21^Waf/Cip1^ to inhibit the activity of cell cycle-dependent kinases (CDKs), preventing the further proliferation of cells [37,38]. When the damage signal enters the S phase, p21^Waf/Cip1^ induced by p53 can bind to the DNA polymerase complex at the replication fork and prevent its activity, inducing cell repair [39]. Additionally, the expression of the well-known universal proapoptotic protein Bax increased significantly in RWPE-1 cells as the avellanin A concentration increased. Direct pharmacological modulation of BAX has long been an attractive target due to the prominent role of BAX in human diseases. Cancer cells often evade apoptosis by overexpressing the antiapoptotic inhibitor BAX [40]; however, small-molecule activators of BAX have been shown to overcome this effect and inhibit cancer growth in animal models [41,42]. These findings suggested that the compound avellanin A affects the cell cycle and blocks cell division and replication through the PI3K-AKT pathway in RWPE-1 cells. 

## 4. Materials and Methods

### 4.1. Fungal Material

The fungus *Aspergillus fumigatus* GXIMD 03099 was isolated from the mangrove plant *Acanthus ilicifolius* L., which was collected from the Beibu Gulf of Guangxi Province in China in July 2020. The strain was deposited at the Institute of Marine Drug Medicine, Guangxi University of Traditional Chinese Medicine, Nanning, China. The fungus was identified according to its morphological characteristics and a molecular biological protocol involving 18S rRNA amplification and sequencing of the ITS region. The sequence data were submitted to GenBank with the accession number ON668102, and the fungal strain was identified as *Aspergillus fumigatus*.

### 4.2. Isolation and Purification

The mycelia and solid rice media were extracted with EtOAc. The organic extract was concentrated in vacuo to yield an oily residue (75.0 g), which was subjected to silica gel column chromatography (CC) (petroleum ether−EtOAc *v*/*v*, gradient 100:0–0:100) to generate ten fractions (Fr. 1–Fr. 10). Fr. 8 (30.1 g) was separated by silica gel CC and eluted with petroleum ether−EtOAc (from 3:1 to 1:1) to afford twelve subfractions (8-1–8-12). Subfractions 8-6 were further purified by using ODS eluted with MeOH−H_2_O *v*/*v* to obtain avellanin A (6.82 mg, *t*_R_ = 22.5 min). The stock material was analysed using ^1^H and ^13^C NMR and high-resolution mass spectrometry prior to use in the studies reported here, and it was found that the material was >98% pure (Supporting Figures S2 and S3 and Table S1).

### 4.3. Cell Culture and Reagents

The RWPE-1 human prostate epithelial cell line was obtained from the Chinese Academy of Sciences (Shanghai, China). The cells were cultured with keratinocyte-SFM (K-SFM, Gibco, Norristown, PA, USA) supplemented with 100 mg/mL penicillin/streptomycin (HyClone, Logan, UT, USA) in an incubator at 37 °C with humidified 5% CO_2_. After 24 h of incubation, the culture media were replaced with fresh media containing 0.5 μM TP (Wako Pure Chemical Industries, Osaka, Japan) in order to induce cell proliferation. Avellanin A was supplemented together within TP-containing media.

### 4.4. Cell Viability Assays

The CCK-8 assay and colony formation assays were used to assess cell viability. The cells were digested by trypsinisation and then seeded in 96-well plates at 5 × 10^3^ cells per well. In the CCK-8 experiment, nine groups were treated with avellanin A separately at concentrations of 0, 0.1, 0.2, 0.3, 0.4, 0.5, 0.6, 0.7, 0.8, 0.9, and 1.0 μM at 37 °C for 24 h, each with eight replicates. Forty-eight hours later, 10 μL of CCK-8 reagent (KeyGEN, Nanjing, China) was added, and the absorbance was measured at 450 nm after incubation for 2–4 h. The absorbance at a wavelength of 450 nm was determined by a microplate reader (Bio Tek Instruments, Bad Friedrichshall, Germany). The CCK-8 assay was performed following the manufacturer’s protocol.

### 4.5. Cell Cycle Assay

To detect the impact of avellanin A on the cell cycle, the RWPE-1 cells were seeded on the 6-well plates treated with avellanin A (0.7 μM) at 37 °C for 24 h. At the end of incubation, cells were trypsinised. The cells (1 × 10^6^) were collected by centrifugation at 1000× *g* for 5 min, washed twice with ice-cold PBS, fixed with cold 70% ethanol, and stored at −20 °C for 24 h. The cells were subsequently centrifuged again, washed twice with cold PBS, incubated with RNase A (0.1 mg/mL) for 1 h at 37 °C, and stained with PI (0.1 mg/mL) for 30 min in the dark. The DNA content was measured by flow cytometry (LSRFortessa, BD, Canton, MA, USA), and the percentage of cells in each phase of the cell cycle was evaluated using ModFit LT version 4.0 software.

### 4.6. Cell Migration Assays

Cell migration assays were performed as follows: The RWPE-1 cells were digested and suspended in serum-free medium. Cells (6 × 10^4^) were seeded in 24-well plates treated with avellanin A (0.7 μM) coated without Matrigel in the upper layer of a migration chamber (Labselect, Hangzhou, China) with 8 µm pore polycarbonate membranes containing 200 µL of FBS-free RPMI-1640 medium (Gibco, Norristown, PA, USA), and the lower layer was supplemented with 500 µL of K-SFM. After 24 h of incubation, unmigrated cells across the membranes were carefully removed with cotton swabs, while cells across the membrane were fixed with 4% paraformaldehyde (Biosharp, Shanghai, China) and then stained with crystal violet dye. Finally, the cells were photographed and counted under an optical microscope. 

### 4.7. Colony Formation Assay

For the colony formation assay, cells (500/well) were cultured in 6-well plates treated with avellanin A (0.7 μM) for 7 days. When the colonies were clearly observed under the microscope, the cells were washed twice with PBS and fixed with 4% paraformaldehyde for 15 min, followed by staining with crystal violet dye (Yunaye, China) for 10 min. The number of cell clones was photographed and statistically analysed.

### 4.8. Transcriptomic Expression Profiling and Bioinformatics Analysis

RWPE-1 cells were treated with avellanin A (0.7 μM) at 37 °C for 48 h in TP-containing media and subsequently collected for transcriptomic analysis. Total RNA was extracted from the RWPE-1 cells using the TRIzol reagent kit (Invitrogen, Carlsbad, CA, USA) and assessed for quality using an Agilent 2100 Bioanalyzer system (Agilent Technologies, Palo Alto, CA, USA). Subsequently, library preparation for sequencing was conducted using the NEBNext Ultra RNA Library Prep Kit for Illumina, following the manufacturer’s instructions. Clustering was carried out on the cBot Cluster Generation System (Illumina, San Diego, CA, USA) using the TruSeq PE Cluster Kit v3-cBot-HS, and sequencing was performed on the Illumina NovaSeq 6000 platform (Gene Denovo Biotechnology, Guangzhou, China).

The obtained clean data were processed by eliminating reads containing adapters and low-quality sequences from the raw data. Alignments of the clean reads with the assembled genome of GRCh38.p13 were performed using HISAT2. The FPKM value of each gene in each sample was determined using featureCounts 2.0.2 software. Genes with a *p*-value < 0.05 and a fold change > 2 were considered to be significantly differentially expressed. All differentially expressed genes (DEGs) were associated with Gene Ontology (GO) terms in the GO database, and the number of genes per term was calculated. The significantly enriched GO terms in DEGs were identified using the hypergeometric test. Additionally, Kyoto Encyclopedia of Genes and Genomes (KEGGs) pathway enrichment analysis was conducted to pinpoint metabolic pathways or signal transduction pathways that were significantly enriched in DEGs compared to the genome-wide background. The calculated *p*-value was adjusted for the false discovery rate (FDR) with a threshold of FDR ≤ 0.05. GO terms and KEGG pathways meeting these criteria were deemed significantly enriched in the DEGs. Then, the genes were searched in the STRING database (25 August 2023) to determine the protein–protein interaction (PPI) relationships. Cytoscape 3.7.2 software was used to construct the PPI network to visualize the relationships between avellanin A and its targets.

### 4.9. Molecular Docking

Molecular docking was performed to evaluate the binding affinity of avellanin A for the cathepsin L protein. The Maestro program in Glide software version 8.1 was used for docking. Human cathepsin L (UniProt ID: P07711) was obtained from the PDB protein database (7 April 2024), while the structures of the avellanin A compounds were downloaded from the PubChem database (7 April 2024). Prior to the docking process, water molecules were removed from the conformation, and the proteins were subjected to hydrogenation. Furthermore, the 3D structure of the target compound was optimised by calculating its 3D conformation at the minimum energy. The interaction between the ligand and the acceptor was analysed, and potential active substances were evaluated based on the score. 

### 4.10. Fluorescence-Based Quantitative PCR

Total RNA from the RWPE-1 cell samples was extracted using TRIzol reagent (Invitrogen, Carlsbad, CA, USA) and then transcribed into cDNA using a reverse transcriptome kit (Applied Biosystems, Foster City, CA, USA). Quantitative PCR was performed on a real-time PCR system. Primers were designed and synthesised by Huiyuan Technology Co., Ltd. (Jinan, China). The primer sequences are shown in Table S2.

### 4.11. Western Blotting 

The Western blot analysis was conducted as follows: Briefly, RWPE-1 cells were treated with avellanin A (0.7 μM) at 37 °C for 48 h in TP-containing media. The cells were washed twice with PBS supplemented with an appropriate amount of RIPA lysis buffer, lysed at 4 °C for 30 min, and centrifuged at 12,000 r/min for 10 min at 4 °C, after which the supernatant contained the total extracted protein. Electrophoresis was performed with a 10% concentration of polyacrylamide gel (SDS-PAGE), and the proteins were transferred to PVDF membranes. The membranes were blocked in 5% milk and incubated with the primary antibodies at 4 °C overnight. Primary antibodies against p-Akt (1:2000, No. 4060), anti-Akt (1:1000, No. 4691), anti-p-PI3K (1:1000, No. 4228), anti-PI3K (1:1000, No. 4257), anti-p53 (1:1000, No. 2527), anti-cyclin D1 (1:1000, No. 55506), anti-p21 Waf1/Cip1 (1:1000, No. 2947), and anti-Bax (1:1000, No. 5023) were purchased from Cell Signaling Technology. Then, the membranes were washed with TBST and incubated with secondary antibodies at 37 °C for two hours. Finally, the bands were visualised by using an Omni-ECL Femto Light Chemiluminescence Kit (Epizyme, Shanghai, China) and imaged by using an Amersham Imager 680 blot gel imager (Cytiva, Marlborough, MA, USA). β-Actin was used as a control, and the test was repeated at least three times.

### 4.12. Statistical Analysis

The data are expressed as the means ± SDs from at least three independent experiments. The unpaired *t* test was used for comparisons between groups. Statistical analyses were carried out by one-way analysis of variance with Bonferroni’s multiple-comparison correction for comparisons among three or more groups. The following labels were used: ns, not significant; *, *p* < 0.05; **, *p* < 0.01; ***, *p* < 0.001; and ****, *p* < 0.0001. GraphPad Prism 10.0 software was used for statistical analysis and graphing.

## 5. Conclusions

In this project, the cyclic pentapeptide compound avellanin A from the *Aspergillus fumigatus fungus* was studied to explore its ability to inhibit RWPE-1 cell proliferation and its molecular mechanism. Avellanin A has a good affinity for cathepsin L, which is involved in the terminal degradation of extracellular matrix components. Avellanin A significantly blocked the ECM–receptor interaction and suppressed the downstream PI3K-Akt signalling pathway. The expression of the genes *COL1A1*, *COL1A2*, *COL5A2*, *COL6A3*, *MMP2*, *MMP9*, *ITGA2*, and *ITGB3* was significantly downregulated after avellanin A intervention. The results confirmed that it not only reduced ITGA2 and FAK/p-FAK protein expression but also inhibited PI3K/p-PI3K and Akt/p-Akt protein expression in the PI3K-Akt signalling pathway. Furthermore, avellanin A upregulated Bax, p21^WAF1/Cip1^, and p53 protein expression and downregulated Cyclin D1 protein expression in TP-induced RWPE-1 cells, leading to cell cycle arrest and inhibition of cell proliferation. This study supports the use of avellanin A as a potential new drug for the treatment of BPH. 

## Data Availability

The data presented in this study are available upon request from the corresponding author.

## Supplementary Materials

The following supporting information can be downloaded at: https://www.mdpi.com/article/10.3390/md22060275/s1, Figure S1: HR-ESI-MS spectrum of avellanin A. Figure S2: ^1^ H NMR (d 4-methanol, 600 MHz) spectrum of avellanin A; Figure S3: ^13^C NMR (d 4-methanol, 150 MHz) spectrum of avellanin A. Figure S4: The original Western blot image of Figure 4C. Figure S5: The original Western blot image of Figure 5. Table S1: ^1^H and ^13^C NMR assignments for avellanin A in DMSO-*d*_6_. Table S2: Sequences of qPCR primers.
