# Supplementary material for: Avellanin A Has an Antiproliferative Effect on TP-Induced RWPE-1 Cells via the PI3K-Akt Signalling Pathway
Source: Mar Drugs. 2024 Jun 13;22(6):275. doi: 10.3390/md22060275 (PMC11205091; doi:10.3390/md22060275)
Supplement: Supplementary file 1 [file marinedrugs-22-00275-s001.zip › marinedrugs-3036305-supplementary.pdf]

Supplementary Information

Avellanin A Has an Antiproliferative Effect on TP-Induced RWPE-1 Cells via the PI3K-Akt Signalling Pathway

List of Supporting Information

Figure S1. HR-ESI-MS spectrum of avellanin A.

Figure S2. ^{1} H NMR (d 4 -methanol, 600 MHz) spectrum of Avellanins A.

Figure S3. ^{13}C NMR (d 4 -methanol, 150 MHz) spectrum of Avellanins A.

Figure S4. The original Western Blot image of Figure 4C.

Figure S5. The original Western Blot image of Figure 5.

Table S1. ^{1}H and ^{13}C NMR assignments for avellanin A in DMSO-$d_{6.}$

Table S2. Sequences of qPCR primers.

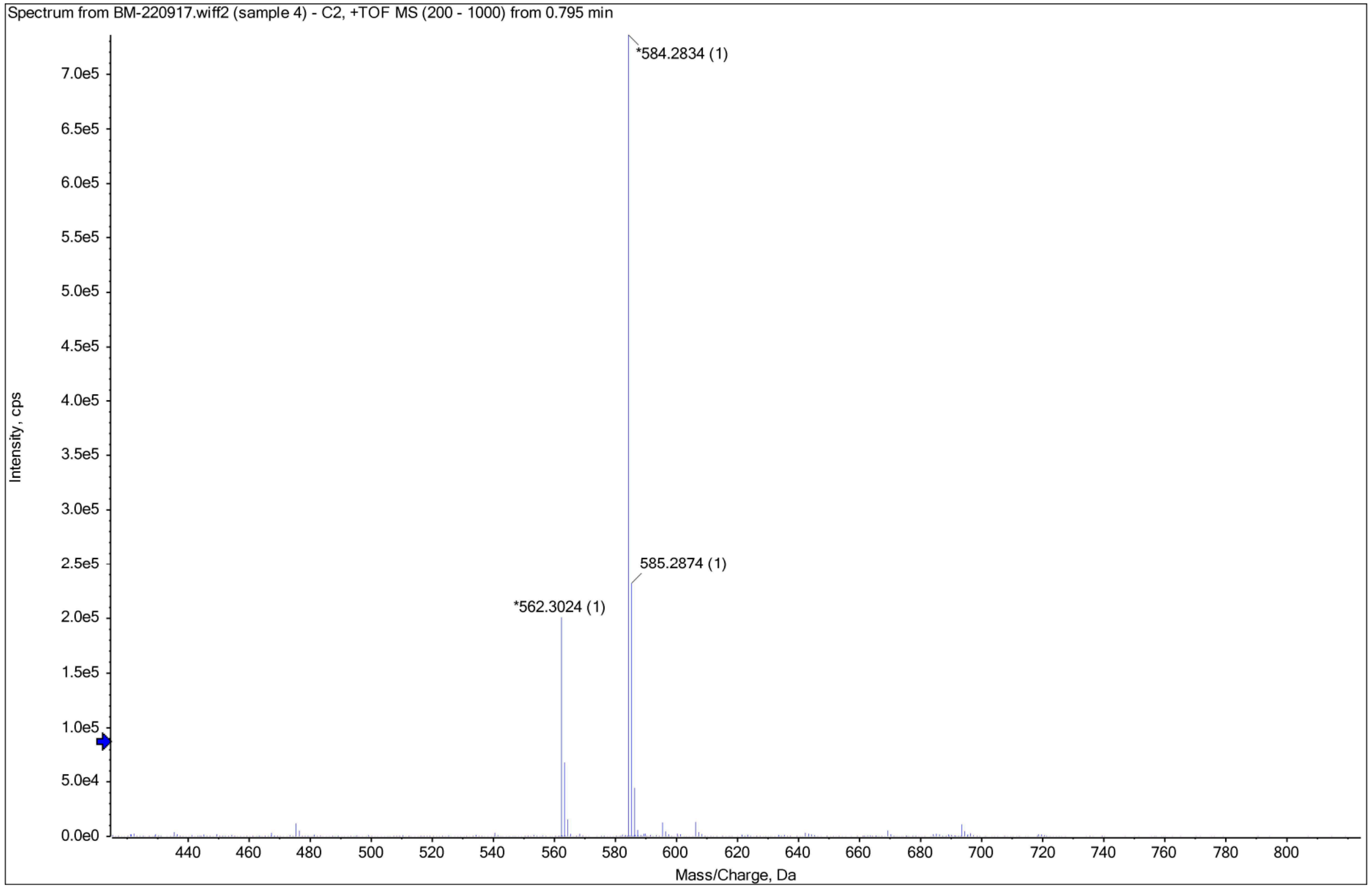

Figure S1. HR-ESI-MS spectrum of avellanin A.

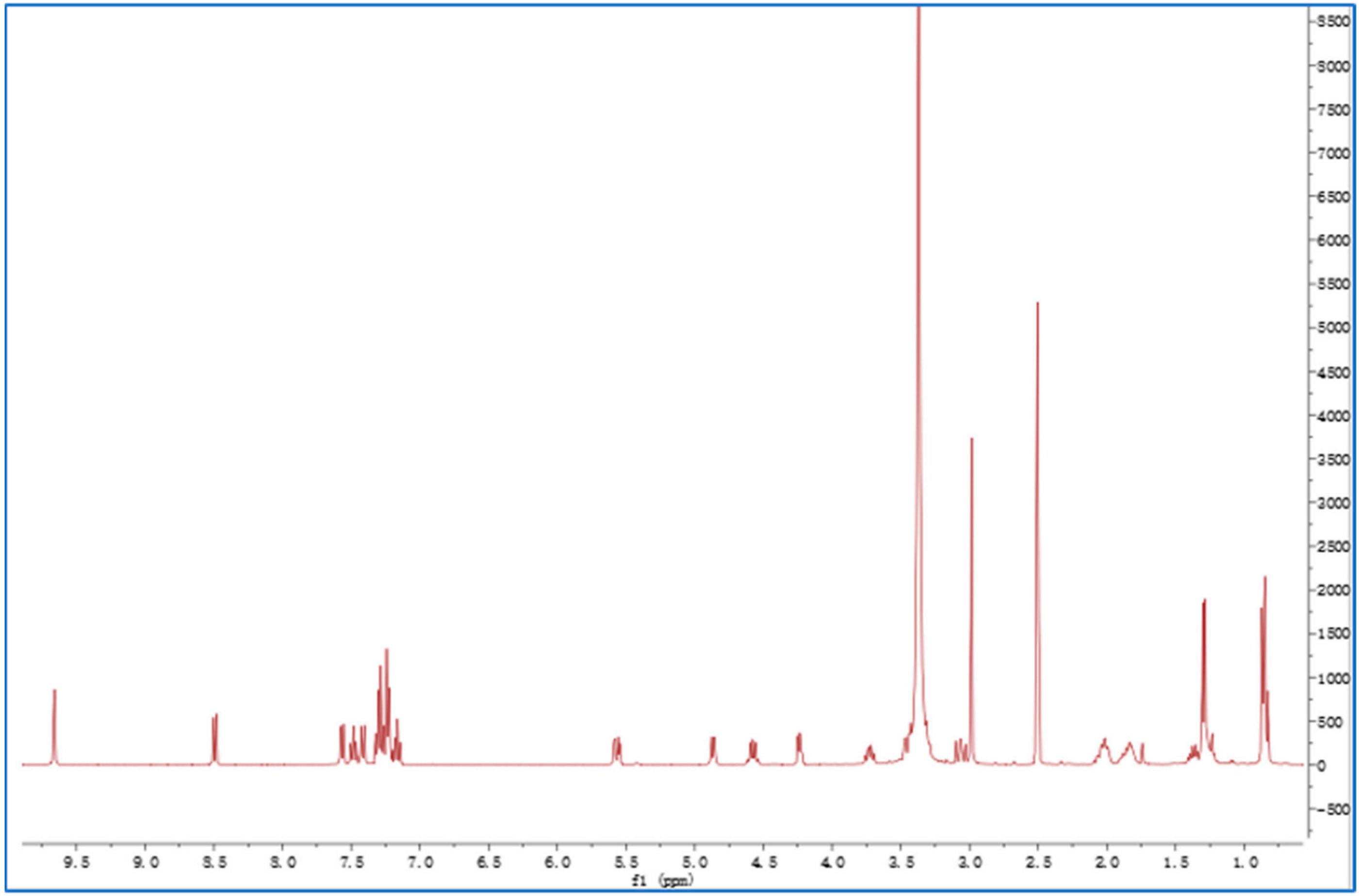

Figure S2. ^{1}H NMR (d 4 -methanol, 600 MHz) spectrum of Avellanins A.

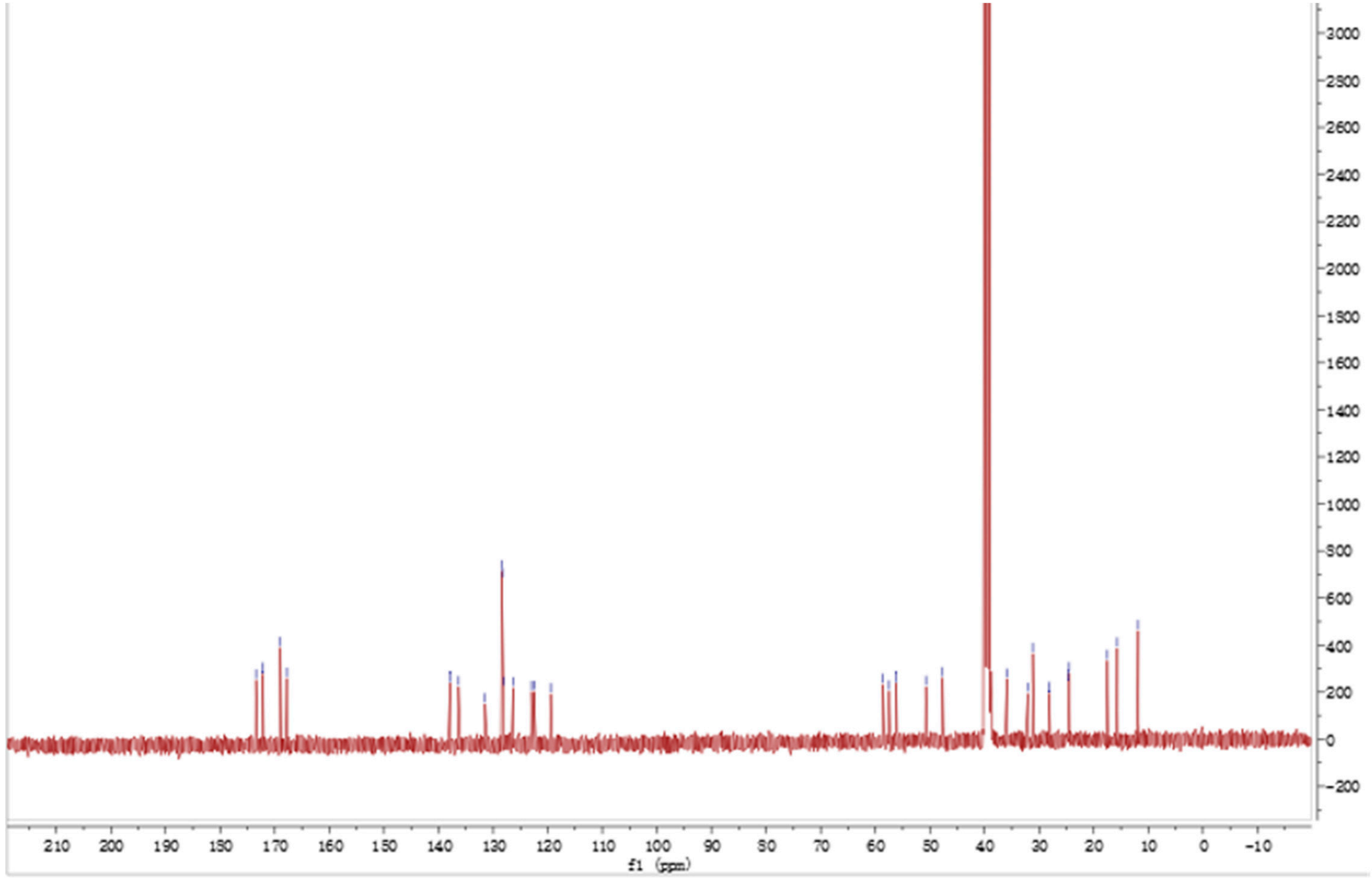

Figure S3. ^{13}C NMR(d 4 -methanol, 150 MHz) spectrum of Avellanins A.

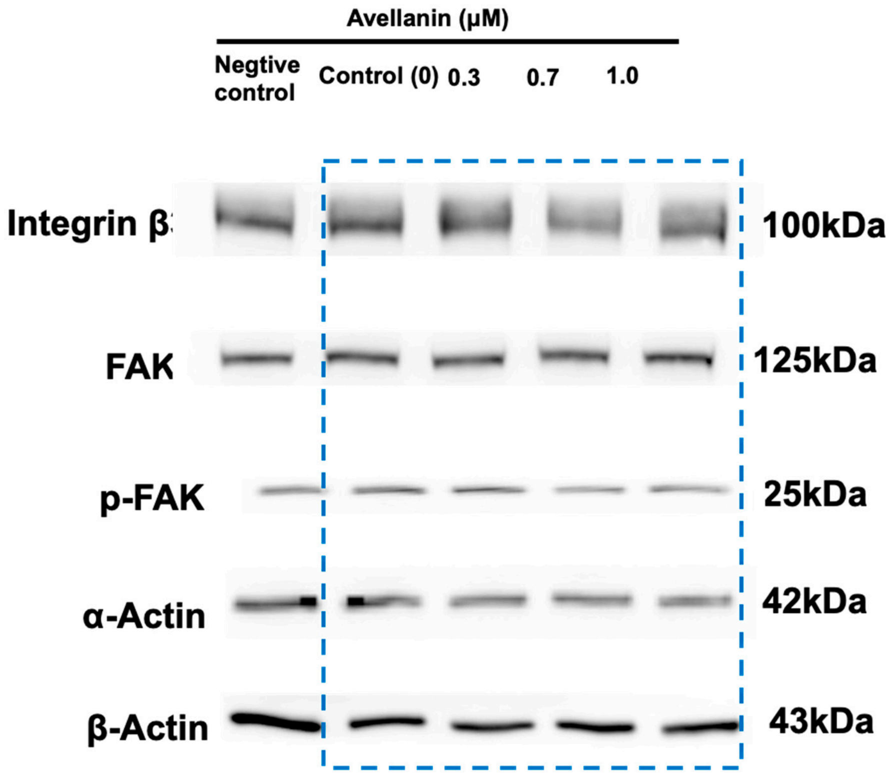

Figure S4. The original Western Blot image of Figure 4C.

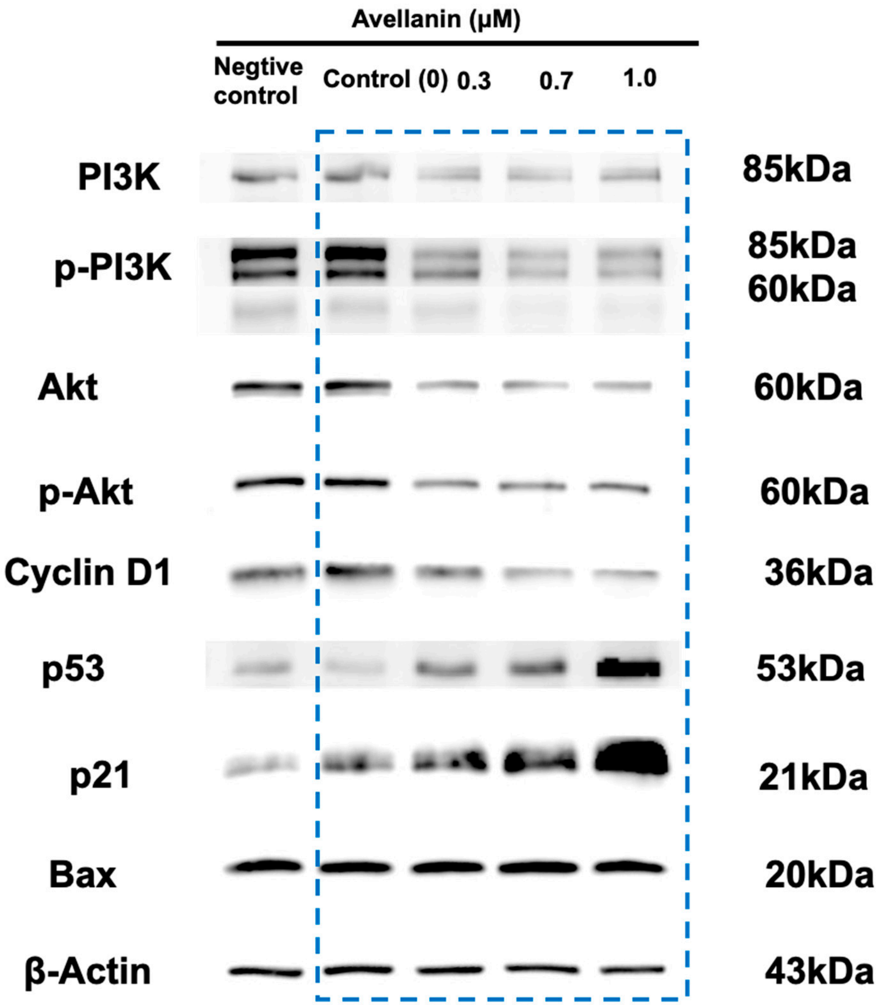

Figure S5. The original Western Blot image of Figure 5.

Table S1. ^{1}H and ^{13}C NMR assignments for avellanin A in DMSO-d_6

Residue	Position	avellanin A (DMSO-d_6)		Reported avellanin A (DMSO-d_6)	
		δ_H, mult. (J in Hz)	δ_C, Type	δ_H, mult. (J in Hz)	δ_C, Type
Ala	1		172.1 (C)		172.0 (qC)
	2	4.58, m	47.6 (CH)	4.72, dd (7.3, 2.6)	48.5 (CH)
	3	1.29, d (7.2)	17.5 (CH_3)	1.52, d (6.8)	18.1 (CH_3)
	NH			7.43, d (7.7)	
Ile	1		168.9 (C)		169.5 (C)
	2	4.24, dd (7.2, 4.0)	57.4 (CH)	4.69, dd (9.4, 3.4)	57.5 (CH)
	3	2.02, m	35.9 (CH)	2.00, m	36.2 (CH)
	4	1.38, dt (12.9, 6.1)	24.5 (CH_2)	1.46, m<br>1.48, m	24.4 (CH_2)
	5	0.85, t (4.8)	11.8 (CH_3)	0.93, t (7.7)	12.2 (CH_3)
	3’	0.86, d (7.2)	15.7 (CH_3)	0.97, d (6.8)	16.3 (CH_3)
Ant	1		168.9 (C)		169.5 (C)
	2		136.3 (C)		136.6 (C)
	3	7.57, dd (8.0, 1.2)	128.1 (CH)	7.32, m	128.2 (CH)
	4	7.16, td (7.6, 0.8)	126.3 (CH)	7.08, t (7.7)	127.3 (CH)
	5	7.48, t (1.6)	131.4 (CH)	7.44, t (7.7)	131.7 (CH)
	6	8.49, d (7.6)	119.4 (CH)	8.45, d (7.7)	121.4 (CH)
	7		122.9 (C)		123.0 (C)
	NH	9.66, s		9.79, s	
Pro	1		173.4 (C)		174.0 (C)
	2	4.87, dd (10.8, 2.0)	56.1 (CH)	4.89, m	56.4 (CH)
	3	2.03, m	28.1 (CH_2)	2.10, m	28.6 (CH_2)

		1.29, m		1.18, m	
	4	1.85, m	24.7 (CH_2)	1.88, m	25.0 (CH_2)
	5	3.31, m<br>3.73, m	50.7 (CH_2)	3.50-3.70, m	51.4 (CH_2)
Phe	1		167.8 (C)		169.7 (C)
	2	5.57, dd (12.4, 4.4)	58.5 (CH)	5.78, dd (12.8, 4.7)	58.5 (CH)
	3	3.44, dd (15.2, 4.0)<br>3.06, dd (15.6, 12.8)	32.0 (CH_2)	3.83, dd (16.2, 4.7)<br>2.97, dd (16.2, 12.8)	33.1 (CH_2)
	4		137.8 (C)		137.1 (C)
	5,9	7.26[d]	128.2 (CH)	7.21[d]	128.2 (CH)
	6,8	7.26[d]	128.4 (CH)	7.30-7.31[d]	128.6 (CH)
	7	7.29[d]	126.3 (CH)	7.24[d]	126.8 (CH)
	NMe	2.98, s	31.1 (CH_3)	3.02, s	31.1 (CH_3)

Table S2. Sequences of qPCR primers

Gene	Forward primer	Reverse primer
COL1A1	5'-CATCTCCATTCTTTCCAGGG-3'	5'-TTCAGTGGTTTGGATGGTG-3'
COL1A2	5'-CATAACCACCACCGCTTAC-3'	5'-TCTTACTCCTGAAGGCTCTAG-3'
COL5A2	5'-CAGGCTCCATAGGAATCAGAG-3'	5'-CAGCATTTCCTGCTTCTCCAG-3'
COL6A3	5'-ACCGGAAGTGTCAATTTCGCA-3'	5'- CAATGACTTGTGTGACAATG-3'
ITGA2	5' -GCCATTGTATATAACATCACA-3'	5'-TTTGTTTTGGTTGCTGACAAT- 3'
ITGB3	5'-CACTCTGCTTCTTCACTTCC-3	5'-TTTACCACTGATGCCAAGAC-3'
MMP2	5'-GAAGAAGAAGATCTCACCAC-3'	5'-GATTGATGCGGTATACGAGG-3'
MMP9	5'-CATCCAGTTTGGTGTCGCG-3'	5'-AAGATGAATGGAACTGGCAG-3'
FGF2	5'-ATCCGTGAACCCCAGGTCC-3'	5'-GGAAGGCGCCGCTGCCGCCAT-3'
VWF	5'CCAGGGCGACGACTTCCTTAC3'	5'TCCGAGCAGGAGCACACGTCG3'
β-actin	5'-CATGTACGTTGCTATCCAGGC-3'	5'-CTCCTTAATGTCACGCACGAT-3'
